# Could targeting viruses be a new hope against neurodegenerative diseases?

**DOI:** 10.1371/journal.pbio.3003669

**Published:** 2026-02-13

**Authors:** Karin M. Danzer, Konstantin MJ Sparrer

**Affiliations:** 1 German Center for Neurodegenerative Diseases (DZNE), Ulm, Germany; 2 Department of Neurology, Ulm University Medical Center, Ulm, Germany; 3 Institute of Molecular Virology, Ulm University Medical Center, Ulm, Germany

## Abstract

Current therapies for neurodegenerative diseases largely manage symptoms and only modestly slow progression. This Perspective highlights emerging evidence that vaccines and antivirals may lower dementia risk by targeting viral triggers, opening new avenues for prevention.

## Teaser

Despite decades of intensive research, our current approaches to treating neurodegenerative diseases only slow down the seemingly inevitable. Could recent data associating vaccinations with reduced risk of dementia offer an unexpected beacon of hope?

Neurodegenerative diseases such as Alzheimer’s disease, Parkinson’s disease, and amyotrophic lateral sclerosis (ALS) are age-associated disorders marked by progressive neuronal loss. The onset and progression of symptoms can vary, but the underlying process is unmistakable: neurons are gradually lost, taking with them a person’s ability to think, move, and behave as they once did [1]. As societies around the world continue to age, the prevalence—and thus the burden—of neurodegenerative diseases is projected to rise steeply. Beyond the personal tragedies surrounding neurodegenerative diseases, they impose an enormous economic burden, driving up healthcare costs and sharply reducing quality of life. Yet, despite progress being made in developing treatments to slow disease progression and alleviate symptoms, the holy grail, an effective preventive measure, has remained seemingly out of reach. Could recent research on the connection between viruses and neurodegenerative diseases provide us with new avenues to combat these conditions in the future? We believe so.

**Fig 1:**
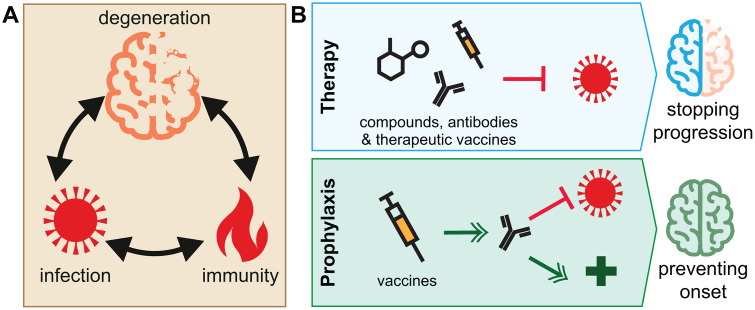
The role of viruses in neurodegenerative diseases and how to target them. **A**. (Viral) infections may directly cause progressive death of neurons. In addition, immune reactions induced by the infection may induce autoimmunity or neuroinflammation that promote the onset and progression of neurodegenerative diseases. **B**. Targeting viral infections using anti-viral compounds, therapeutic vaccines, or modulating the immune system to mitigate infection-induced immunopathogenesis may halt the progression of neurodegenerative diseases (top panel). Prophylactic strategies against viruses such as vaccines (and their adjuvants) may protect against infection or stimulate the immune system to prevent the onset of neurodegenerative diseases (bottom panel). These approaches and modes of action are not mutually exclusive.

Mounting evidence suggests that viral infections may contribute to the onset and progression of neurodegenerative diseases [2] (Fig 1A). Neuroinvasive viruses may cause direct damage to neurons by altering proteostasis, inducing oxidative stress and mitochondrial dysfunction. In addition, chronic and aberrant activation of the central nervous system’s innate immune defenses during infections may drive neurodegenerative disease [2]. For example, studies from almost three decades ago suggested that the neuroinvasive Herpes Simplex Virus 1 (HSV-1) may be associated with Alzheimer’s disease [2,3]. Molecular studies further supported this link by showing that HSV-1 promotes amyloid precursor protein processing, tau phosphorylation, and neuroinflammation [4]. However, robust epidemiological, mechanistic, and serological studies confirming this association are still missing [5]. Other viruses may not reach the central nervous system but instead disturb immune responses in the long run, including triggering the production of autoantibodies that quietly harm neurons over time. Notably, this mechanism has been suggested as one of the underlying reasons for the association between Epstein–Barr Virus and multiple sclerosis; a similar mechanism could also promote neurodegenerative diseases.

Viral infections thus may appear to add fuel to the multifactorial fire of neurodegeneration, whether by direct damage or via the immune system (Fig 1A). To us, this insight may also give hope for future preventive strategies, as targeting infections could reduce the risk of getting neurodegenerative diseases. We already have effective tools available to reduce the burden of some infectious diseases—anti-viral molecules and vaccines. Depending on the relationship between the virus and neurodegenerative disease, vaccines could potentially prevent the onset of neurodegeneration or work therapeutically to prevent the reactivation of an existing pathogen, whereas anti-viral medication after diagnosis with a neurodegenerative disease could potentially slow down progression (Fig 1B).

Supporting these notions, several recent high-profile retrospective studies have suggested that vaccines could indeed help reduce the risk of developing dementias [6–8]. This was initially convincingly demonstrated in a “natural experiment” that showed that vaccination against Varicella Zoster Virus (VZV) with the live attenuated vaccine Zostavax reduced the probability of a new dementia diagnosis by 20% [6]. The currently more widely used VZV vaccine, Shingrix, is a recombinant subunit vaccine containing the VZV surface glycoprotein E, and was also reported to be associated with a 17% increase in diagnosis-free time [8]. The impact of a third VZV vaccine, Varivax, primarily used to prevent chickenpox, is currently unclear, as the first cohort of vaccinated children is not yet of sufficient age to measure an effect. However, it is not just VZV; recent research has suggested that other vaccines, such as those against Respiratory Syncytial Virus or Hepatitis A, are also associated with a reduced risk of dementia [9,10].

So what exactly is happening? One explanation is straightforward: vaccines prevent viral infections, and fewer infections mean less risk of brain damage. In line with this idea, a recent study demonstrated that repeated reactivation of latent Herpes Zoster Virus, but not a single acute episode, was associated with an increased risk of dementia [11]. Another intriguing possibility is that vaccines train or “tune up” the immune system in ways that make it more resilient, thereby indirectly protecting the brain. Indeed, the protective impact of the VZV vaccine was much more pronounced in females, who tend to have better vaccine responses than males anyway, hinting to an immune-mediated phenotype [6]. The idea that mere immune stimulation is sufficient to induce protection is attractive. Taking a different approach, a recent publication proposed that the adjuvant AS01 could be responsible for the protective effect [9]. However, as both adjuvanted and non-adjuvanted shingles vaccines seem to provide protection, the evidence indicates that this cannot be solely due to AS01 [6,7]. In the end, vaccines could be working in multiple ways, keeping viruses at bay while also providing beneficial stimulation to the immune system. And it is not just vaccines: some studies report that anti-viral medications may also reduce dementia risk. For example, epidemiological studies have suggested that use of anti-viral drugs that target Herpes viruses, such as acyclovir, reduce the probability of developing Alzheimer’s disease [5,10].

If vaccines or anti-viral treatments truly keep the brain healthier even during old age, the implications for public health could be enormous. But several open questions remain to be answered and future research is clearly needed. Currently, it is the connection between dementias and viral infections that has mostly been studied. What about motor neuron diseases or movement disorders? Are certain viruses linked more strongly to specific neurodegenerative diseases? Is more than one virus or a whole family of viruses linked to a distinct neurodegenerative disease? Could repeated, carefully timed “immune boosts” delay detrimental immune aging? And could vaccines against bacteria or other pathogens offer similar benefits? We believe that untangling the connections between pathogens, immunity, and neurodegeneration will be essential for turning today’s observations into tomorrow’s therapies. To do so will require prospective and mechanistic studies that explore the neuroprotective effects of anti-viral measures.

Vaccines have long been seen as shields against infectious diseases. While the benefits might take decades to become visible at the population level, the idea that a vaccine could have the added benefit of preserving memory, independence, and quality of life is profoundly motivating. In fact, vaccines may turn out to be a rare double win: protecting us from dangerous infections while also lowering our risk of developing neurodegenerative diseases. We hope that this premise can positively influence how society views and accepts scientifically proven anti-viral measures including vaccines. In the future, this may inspire research into vaccines for viruses where no or only insufficient prophylactic measures are currently available. We do not yet know whether this line of investigation will lead to a true breakthrough in healthy aging—one that enables us to substantially reduce the risk of developing a neurodegenerative disease before it begins. But we hope that future research will allow a refined understanding of the connection between viruses and neurodegenerative diseases, and with that open up direly needed new perspectives for therapy and prophylaxis.

## Abbreviations

ALS: amyotrophic lateral sclerosis
HSV-1: Herpes Simplex Virus 1
VZV: Varicella Zoster Virus
